# Epigenetic aging and fecundability: the Norwegian Mother, Father and Child Cohort Study

**DOI:** 10.1093/humrep/deae242

**Published:** 2024-10-22

**Authors:** Lise Andrea Arge, Yunsung Lee, Karoline Hansen Skåra, Mikko Myrskylä, Cecilia Høst Ramlau-Hansen, Siri Eldevik Håberg, Maria Christine Magnus

**Affiliations:** Centre for Fertility and Health, Norwegian Institute of Public Health, Oslo, Norway; Department of Community Medicine and Global Health, Institute of Health and Society, University of Oslo, Oslo, Norway; Centre for Fertility and Health, Norwegian Institute of Public Health, Oslo, Norway; Centre for Fertility and Health, Norwegian Institute of Public Health, Oslo, Norway; Max Planck Institute for Demographic Research, Rostock, Germany; Helsinki Institute for Demography and Population Health, University of Helsinki, Helsinki, Finland; Max Planck-University of Helsinki Center for Social Inequalities in Population Health, Rostock, Germany; Department of Public Health, Aarhus University, Aarhus, Denmark; Centre for Fertility and Health, Norwegian Institute of Public Health, Oslo, Norway; Department of Global Public Health and Primary Care, University of Bergen, Bergen, Norway; Centre for Fertility and Health, Norwegian Institute of Public Health, Oslo, Norway

**Keywords:** epigenetic age acceleration, epigenetic age deceleration, fecundability, fertility, Norwegian Mother Father and Child Cohort Study

## Abstract

**STUDY QUESTION:**

Is there an association between male or female epigenetic age acceleration (EAA) or deceleration (EAD) and fecundability?

**SUMMARY ANSWER:**

We do not find compelling evidence of an association between EAA or EAD and fecundability.

**WHAT IS KNOWN ALREADY:**

Prior research has shown that female accelerated epigenetic aging is associated with unfavorable clinical fecundity outcomes and use of *in vitro* fertilization, and that epigenetic aging in sperm cells is associated with unfavorable sperm parameters. Studies of epigenetic aging and fecundability among individuals who conceive naturally are lacking.

**STUDY DESIGN, SIZE, DURATION:**

This study is based on the Norwegian Mother, Father and Child Cohort Study (MoBa), a population-based pregnancy cohort which recruited pregnant couples between 1999 and 2008. We used data from 1657 couples (women and men) with planned naturally conceived pregnancies and available blood samples.

**PARTICIPANTS/MATERIALS, SETTING, METHODS:**

Methylation levels were measured in DNA from blood samples taken recruitment (at ∼18 gestational weeks) from pregnant women and their partners using the Illumina Methylation EPIC Array. To obtain a measure of EAA/EAD, we performed a linear regression of each of seven different established epigenetic biomarkers (DNAmAge by Horvath, DNAmAge by Hannum *et al.*, PhenoAge by Levine *et al.*, DunedinPoAm by Belsky *et al.*, DunedinPACE by Belsky *et al.*, DNAmTL by Lu *et al.*, and GrimAge by Lu *et al.*) against chronological age. We fitted proportional probability regression models to obtain fecundability ratios (FRs) for each standard deviation increase in epigenetic aging, and obtained crude and adjusted (for body mass index, smoking, and education level) estimates. Results were evaluated at a false discovery rate (FDR) of 5%. We evaluated all models for non-linear associations using categories of epigenetic age where appropriate.

**MAIN RESULTS AND THE ROLE OF CHANCE:**

Although the DunedinPACE clock in males demonstrated slightly increasing fecundability with increasing EAA (adjusted FR 1.05 per one standard deviation increase in EAA, 95% CI 1.00–1.10), this was not robust when evaluated at an FDR of 5%. We found evidence of non-linearity between biological aging and fecundability in two models in females and three models in males, but non-linear associations were weak and conflicting.

**LIMITATIONS, REASONS FOR CAUTION:**

As MoBa is a pregnancy cohort, our findings may not be generalizable to all couples attempting conception. Fecundability is a couple-level measure, and any impacts of epigenetic aging in each partner may be obscured by effects of the other partner.

**WIDER IMPLICATIONS OF THE FINDINGS:**

Our findings contrast with those of prior studies, which have indicated an association between EAA and unfavorable clinical fertility outcomes in populations using fertility treatments, possibly due to less important effects of epigenetic aging among couples who conceive naturally. More research is needed on the association between blood-based EAA and clinical fertility parameters in both sexes.

**STUDY FUNDING/COMPETING INTEREST(S):**

The study was supported by the Research Council of Norway through its Medical Student Research Program funding scheme (project number 271555/F20), its Centres of Excellence funding scheme (project number 262700), and a grant from the Women’s Health Program (320656). Co-funding was also received from the Strategic Research Council (SRC), FLUX consortium, decision numbers 345130 and 345131; the National Institute on Aging (R01AG075208); grants to the Max Planck—University of Helsinki Center from the Max Planck Society (decision number 5714240218), Jane and Aatos Erkko Foundation, Faculty of Social Sciences at the University of Helsinki, and Cities of Helsinki, Vantaa, and Espoo; and the European Research Council; and the European Research Council (ERC Synergy, BIOSFER, grant number 101071773, and the Horizon 2020 research and innovation program, grant number 947684). The authors declare no conflicts of interest.

**TRIAL REGISTRATION NUMBER:**

N/A.

## Introduction

Fecundability can be defined as the probability of conception within a given menstrual cycle (Weinberg and Wilcox, 2008). It is a couple-level measure that can be influenced by an array of physiological and environmental factors, and female chronological age is among the most well-established predictors (George and Kamath, 2010). Fecundability declines with advancing female age, most markedly from around 35 years (Baird *et al.*, 2005; George and Kamath, 2010; Wesselink *et al.*, 2017). A decrease in fecundability is also seen with advancing age among men, although the decline is not as distinct as that seen in women (Matorras *et al.*, 2011; Jimbo *et al.*, 2022). Despite an established age-related decline in fecundability in both sexes, many individuals retain high fecundability at more advanced ages, indicating that differences in underlying biological aging may play a role in reproductive capability.

Epigenetic clocks based on DNA methylation measurements have been used to estimate differences in biological aging. Among the first epigenetic clocks developed were the blood-based Hannum *et al.* clock (Hannum *et al.*, 2013) and the pan-tissue Horvath clock (Horvath, 2013). Several later clocks, among them PhenoAge by Levine *et al.* (2018) and DNAmTL (Lu *et al.*, 2019b) and GrimAge (Lu *et al.*, 2019a) by Lu *et al.* have incorporated clinical measures and aim to predict morbidity, mortality and lifespan. The clocks DunedinPoAm (Belsky *et al.*, 2020) and DunedinPACE (Belsky *et al.*, 2022) by Belsky *et al.* were constructed to predict the pace of aging using longitudinal data of different biomarkers related to the integrity of several organ systems.

Several studies have explored the association between epigenetic aging and clinical aspects of fecundity in females. In assisted reproductive technology (ART) populations, accelerated epigenetic aging is associated with lower anti-Müllerian hormone (AMH) values (Monseur *et al.*, 2020; Knight *et al.*, 2022), oocyte yield (Hanson *et al.*, 2020; Monseur *et al.*, 2020; Knight *et al.*, 2022), antral follicle count (Knight *et al.*, 2022), and number of successfully fertilized embryos (Knight *et al.*, 2022). A modestly higher epigenetic age has been demonstrated in *in vitro* fertilization (IVF) patients who did not achieve a live birth compared with those who did (Li Piani *et al.*, 2022). These studies are relatively small (31–181 participants) and lack a non-ART control group. Couples undergoing ART are a highly selected population with a generally lower fecundity than average, so results from such populations are not necessarily generalizable to couples who conceive naturally. Additionally, the process of ART may itself accelerate epigenetic aging, introducing potential for reverse causality when exploring this association in an ART-only population.

In a larger 2022 study, Lee *et al.* (2022) compared the epigenetic age acceleration (EAA) and deceleration (EAD) of mothers and fathers in 894 IVF couples and 1000 couples who conceived naturally. They found that IVF mothers had a slightly faster pace of aging than non-IVF mothers according to the DunedinPoAm clock but found no difference in fathers.

Aside from the Lee *et al.* study, there is to our knowledge no research on the association between blood DNA methylation based EAA/EAD and similar clinical aspects of male fertility. However, some research exists on DNA methylation in sperm. One study found shorter time-to-pregnancy (TTP) in participants with DNA methylation-based EAD in sperm (Pilsner *et al.*, 2022). Another found higher sperm count, sperm concentration, sperm motility, and pregnancy rates after intra-cytoplasmatic sperm injection (ICSI) in men with higher global DNA methylation in sperm (Roshdy *et al.*, 2020).

Despite the well-established correlation between chronological age and fecundability, studies exploring the association between EAA/EAD and fecundability are lacking, particularly in couples who conceive naturally. Our objective was therefore to investigate the association between EAA/EAD and fecundability in couples with naturally conceived pregnancies participating in the Norwegian Mother, Father and Child Cohort Study (MoBa), using seven different DNA methylation based epigenetic clocks (Hannum *et al.*, 2013; Horvath, 2013; Levine *et al.*, 2018; Lu *et al.*, 2019a,b; Belsky *et al.*, 2020, 2022).

## Materials and methods

### Study population

The Norwegian Mother, Father and Child Cohort Study (MoBa) is a population-based pregnancy cohort study conducted by the Norwegian Institute of Public Health (Magnus *et al.*, 2016). Participants were recruited from all over Norway from 1999 to 2008. For 41% of the pregnancies, the women consented to participation. The cohort includes approximately 114 500 children, 95 200 mothers, and 75 200 fathers. The establishment of MoBa and initial data collection was based on a license from the Norwegian Data Protection Agency and approval from The Regional Committees for Medical and Health Research Ethics. The MoBa cohort is currently regulated by the Norwegian Health Registry Act. The current study was approved by The Regional Committees for Medical and Health Research Ethics of South/East Norway (2017/1362). Blood samples were obtained from both parents during pregnancy and from mothers and children (umbilical cord) at birth (Rønningen *et al.*, 2006; Paltiel *et al.*, 2014).

**Figure 1.**
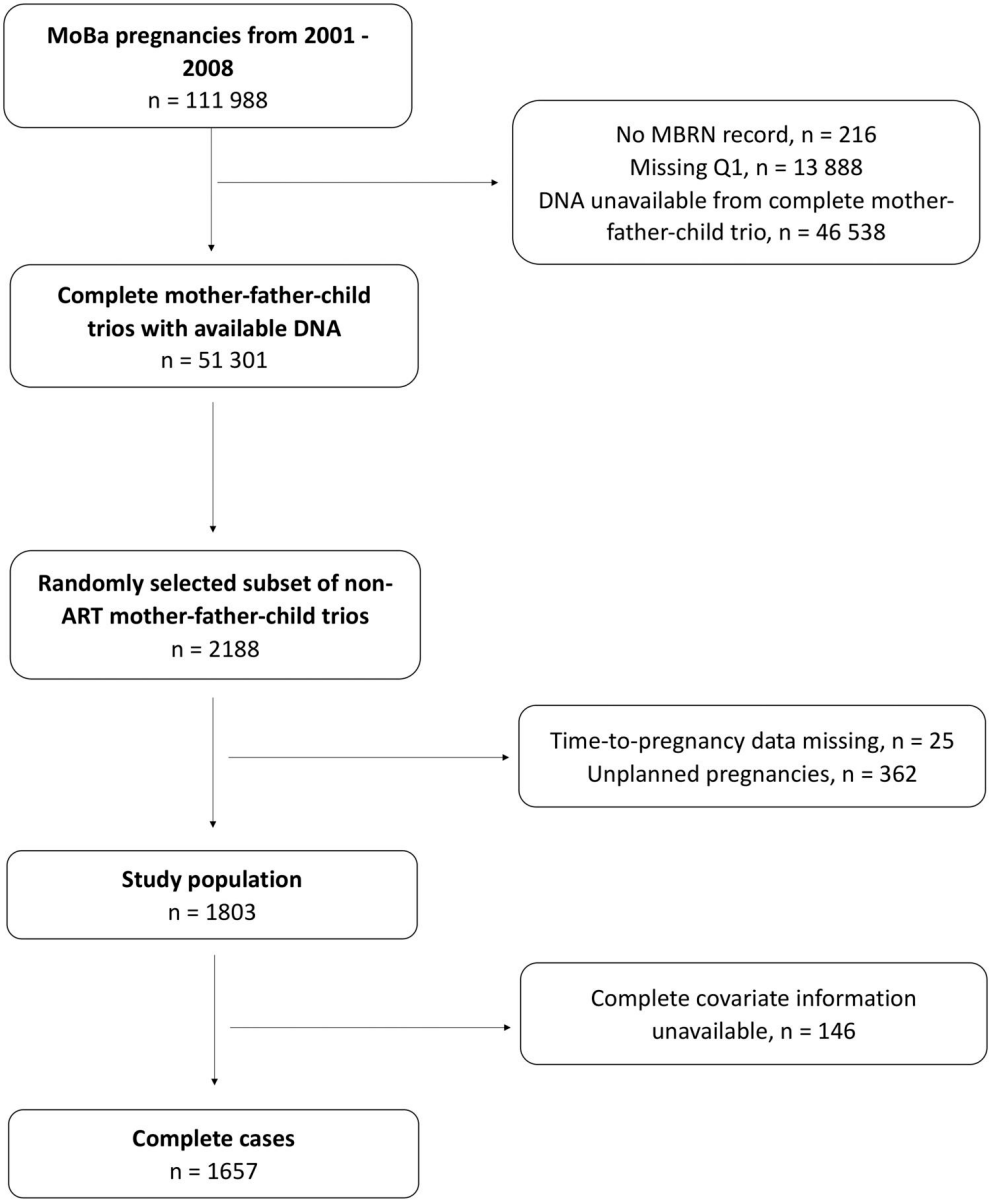
**Inclusion criteria.** MoBa, Norwegian Mother, Father and Child Cohort Study; MBRN, Medical Birth Registry of Norway; ART, assisted reproductive technologies.

This study focused on couples (women and men) in the MoBa who had available measures of DNA methylation from blood samples collected from the pregnant women and their partners at the time of recruitment into the cohort (∼18 gestational weeks). A subset of these couples were randomly selected for estimation of epigenetic aging parameters, as described previously (Lee *et al.*, 2022). Couples with unplanned pregnancies and pregnancies where time-to-pregnancy (TTP) data were missing were excluded, as planning data is necessary to calculate fecundability. Of the 1803 eligible couples, 1657 had complete information on all covariates and were included in the main analyses. The selection of couples for the main analysis are shown in Fig. 1. In a sensitivity analysis including non-planners, 330 non-planners with complete covariate information were included.

### Fecundability

Fecundability was calculated using self-reported information from participants. At recruitment, women were asked whether their pregnancy was planned. For planned pregnancies, they were asked to report for how long they had tried to conceive (TTP) in months (<1 month/1–2 months/3 months or more/exact number of months if 3 months or more). TTP was set to 1, 2, and 3 months for the first three categories, and the exact number of months was used where reported. Where information on cycle length was reported (94.1%), we used menstrual cycles as the unit of analysis by dividing TTP by the woman’s cycle length in days and multiplying by 28. In couples where cycle length was not reported, we used months as the unit of analysis.

### DNA methylation and epigenetic age acceleration

The measurement of blood-based DNA-methylation, calculation of epigenetic age, and associated quality control procedures are described in detail by Lee *et al.* (2022). Briefly, methylation was measured using the Illumina Infinium MethylationEPIC Array (Pidsley *et al.*, 2016). After a quality control procedure, 770 586 autosomal probes were identified and used to calculate epigenetic age. Epigenetic age biomarkers were obtained by averaging DNAm levels across cell types at selected CpGs.

Seven epigenetic clocks were analyzed in this study. Hannum *et al.*’s epigenetic clock (Hannum *et al.*, 2013), developed based on blood-derived DNAm data, is widely used as a baseline for comparisons with other epigenetic clocks. Horvath *et al.* (Horvath, 2013) is pan-tissue clock trained on DNAm data from multiple tissues, making it particularly valuable for assessing reproductive aging, which involves various tissues. Levine *et al.*’s PhenoAge (Levine *et al.*, 2018) and Lu *et al.*’s GrimAge (Lu *et al.* 2019a) have demonstrated solid associations with cardiovascular disease, mortality and morbidity, which are closely linked to reproductive health. Lu *et al.*’s DNAmTL, which estimates telomere length as a biomarker of cellular aging and replicative potential, is relevant for assessing reproductive capacity such as ovarian reserve or sperm production. Belsky *et al.*’s DunedinPoAm (Belsky *et al.* 2020) and DunedinPACE (Belsky *et al.*, 2022) were included due to their ability to capture the pace of functional decline in adults of reproductive age, especially 38–45 years.

For the DNAmAge, PhenoAge, and GrimAge clocks, the calculated epigenetic age biomarkers estimate epigenetic age (in years) at sampling time (Hannum *et al.*, 2013; Horvath, 2013; Levine *et al.*, 2018; Lu *et al.*, 2019a). For the DNAmTL clock, they estimate telomere length (in kilobases) at sampling time (Lu *et al.*, 2019b). For DunedinPoAm and DunedinPACE, they reflect the pace of aging (in years) over the past 10–15 years at sampling time (Belsky *et al.*, 2020, 2022). Despite these varying dynamics, the different estimates of epigenetic age were treated in the same way in the subsequent analyses, as they all reflect peer differences in aging or aging speed. Any associations may therefore also be interpreted similarly for the seven clocks.

To obtain a measure of EAA/EAD, we performed a linear regression of each biomarker against chronological age. The resulting residual terms were standardized into *Z*-scores, so that negative *Z*-scores denote EAD and positive *Z*-scores denote EAA.

### Covariates

We used information on covariates collected from MoBa questionnaires at the time of recruitment. These included body mass index (pre-pregnancy for women, categories <18.5/18.5–24.9/25–29.9/≥30 kg/m^2^), smoking status (no/sometimes/daily) and highest completed or ongoing education (less than high school/high school/up to 4 years of college/more than 4 years of college). For smoking status in women, we considered self-reported smoking status three months prior to pregnancy. For smoking status in men, we considered self-reported smoking status six months prior to pregnancy where reported; if missing, we considered partner’s smoking status as reported by the women (6.6%).

### Statistical analyses

A fecundability ratio (FR) is a risk ratio that denotes the ratio of per-cycle probability of conception in an exposed group relative to a reference group, so that an FR below 1 denotes a lower fecundability (Weinberg and Wilcox, 2008; Basso *et al.*, 2021). We estimated FRs separately for each epigenetic clock using proportional probability regression models (Weinberg and Wilcox, 2008; Basso *et al.*, 2021), with menstrual cycles as the unit of analysis and achievement of pregnancy during the given cycle as the outcome. We censored at six cycles, both to reduce the impact of couples taking longer to conceive being older at the time of blood sampling, and because TTP data is more reliable when TTP is shorter (Radin *et al.*, 2015). Because underlying fecundability is constant over a short period of time, censoring at different time points yields similar results, as the ratio of those who conceive every cycle is approximately the same.

*Z*-scores from the regression of epigenetic against chronological age were treated as the exposure variable. The resulting FR represents the change in fecundability for one standard deviation increase in EAA. To account for multiple testing, we evaluated the results at a false discovery rate (FDR) of 5%. This is performed by ranking result by *P*-value in ascending order. Each *P*-value is then assessed at a corrected *α* level = 0.05 × rank/*n*, where *n* is the total number of tests (7, accounting for seven separate epigenetic aging models).

We tested each model for non-linearity by including *Z*-scores as a linear and squared term and evaluating the significance of the squared term. To explore the association categorically, we categorized participants according to their epigenetic aging profile: very decelerated aging (*Z*-score <−1.5), moderately decelerated aging (*Z*-score −1.5 to −0.5), neither accelerated nor decelerated aging (*Z*-score −0.5–0.5), moderately accelerated aging (*Z*-score 0.5–1.5) and very accelerated aging (*Z*-score >1.5). The FRs from these analyses represent the ratio of the fecundability in each epigenetic aging category relative to participants with neither accelerated nor decelerated aging, who were treated as the reference group.

All statistical analyses were carried out in Stata version 17 (StataCorp, TX, USA).

### Sensitivity analyses

We conducted several sensitivity analyses to explore the robustness of our findings.

We explored the effect of different combinations of partner epigenetic aging profiles by dividing couples into nine groups according to epigenetic aging profile: both partners had no EAA/EAD (reference group), woman had EAA and man had neither, man had EAA and woman had neither, both partners had EAA, woman had EAD and man had neither, man had EAD and woman had neither, both partners had EAD, woman had EAA and man had EAD, and man had EAA and woman had EAD. The thresholds for decelerated and accelerated aging in this analysis were set to *Z* = −0.5 and *Z* = 0.5 respectively, as thresholds of *Z* = 1.5 yielded too small groups for analysis.

To explore whether epigenetic effects on aging differed according to chronological age, we conducted analyses stratified by chronological age. We used the median age in women, 30 years, as a cutoff. We also explored additional adjustment for parity, chronological age and blood sample cell type composition. Cell-type composition was estimated from blood-derived DNAm data using the minfi R package and the FlowSorted.Blood.EPIC reference dataset (Salas *et al.*, 2018). We used the estimateCellCounts2 function with preprocess Noob for background correction and an iterative algorithm for Identifying Optimal Libraries (IDOL) for probe selection. Finally, we explored the effect of excluding non-planners in the main analysis by including them in a sensitivity analysis, setting their TTP to 1, as they are assumed to be a highly fecund group.

## Results

### Participant characteristics

Participant characteristics are detailed in Table 1. Mean female and male age were 30.3 (SD 4.6) and 32.8 (SD 5.4), respectively. The distribution of background characteristics was similar to that observed for all women and men from complete mother-father-child trios in MoBa, shown in Supplementary Table S1. Overall, the correlation between the estimates from the different clocks evaluated were modest (pairwise Pearson correlation coefficients are shown in Supplementary Tables S2 and S3). The distribution of chronological age and biomarkers of predicted epigenetic age (expressed as predicted epigenetic age, pace of aging or estimated telomere length) for women and men are represented graphically in Supplementary Figs S1 and S2, respectively.

**Table 1.** Characteristics of female and male participants (n = 1803 couples).

Characteristics	Women	Men
**Age, mean (SD)**	30.3 (4.6)	32.8 (5.4)
**Education (%)**		
Less than high school	3.7	6.4
High school	25.4	37.5
College, up to 4 years	43.0	29.8
College, more than 4 years	27.8	23.9
Missing	0.2	2.4
**Smoking (%)**		
No	72.8	71.1
Sometimes	10.8	11.5
Daily	15.8	17.3
Missing	0.7	0.1
**Body mass index (pre-pregnancy for females) (%)**		
Underweight (<18.5)	2.5	0.0
Normal weight (18.5–24.9)	64.6	40.9
Overweight (25–29.9)	21.9	45.1
Obese (≥30)	9.5	10.2
Missing	1.6	3.8
**Parity (%)**		
0	47.8	
1	36.8	
2	13.6	
≥3	1.8	

### Women

Unadjusted estimates in two models indicated a slight linear decrease in fecundability with increasing EAA, with FRs of 0.95 (0.91–0.99) and 0.96 (0.91–1.00) in the DunedinPACE and GrimAge models respectively (Table 2). This corresponds to a 4–5% decrease in the monthly probability of conception per standard deviation increase in EAA. However, these results were not robust to adjustment for covariates; none of the adjusted results demonstrated a significant linear association between epigenetic aging and fecundability in women.

**Table 2.** Unadjusted and adjusted fecundability ratios according to female epigenetic aging.

		Fecundability ratio	95% confidence interval	*P*, linear trend	*P*, non-linearity
**DNAmAge (Horvath)**	**Unadjusted**	0.99	0.94–1.03	0.506	0.116
	**Adjusted**	0.99	0.94–1.03	0.544	0.233
**DNAmAge (Hannum *et al.*)**	**Unadjusted**	0.98	0.93–1.02	0.312	**<0.001**
	**Adjusted**	0.99	0.94–1.03	0.585	**<0.001**
**PhenoAge (Levine *et al.*)**	**Unadjusted**	0.96	0.91–1.00	0.053	**0.030**
	**Adjusted**	0.97	0.92–1.01	0.173	**0.000**
**DunedinPoAm (Belsky *et al.*)**	**Unadjusted**	0.97	0.93–1.01	0.146	0.448
	**Adjusted**	0.98	0.94–1.03	0.402	0.496
**DunedinPACE (Belsky *et al.*)**	**Unadjusted**	**0.95**	**0.91–0.99**	**0.025**	0.078
	**Adjusted**	0.98	0.94–1.03	0.444	0.139
**DNAmTL (Lu *et al.*)**	**Unadjusted**	0.99	0.94–1.03	0.514	0.276
	**Adjusted**	0.97	0.93–1.02	0.233	0.236
**GrimAge (Lu *et al.*)**	**Unadjusted**	**0.96**	**0.91–1.00**	**0.046**	0.064
	**Adjusted**	0.97	0.92–1.02	0.204	0.066

Adjusted for pre-pregnancy body mass index, smoking, and highest completed or ongoing education. Fecundability ratios per one standard deviation increase in epigenetic age acceleration.Statistically significant results at *α* = 0.05 are highlighted in bold.

**Figure 2.**
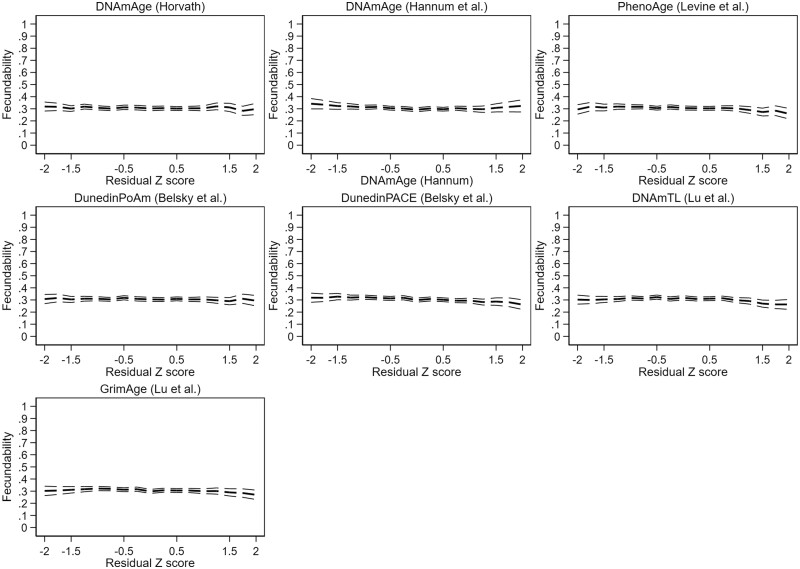
**Unadjusted couple fecundability according to female epigenetic aging profile.** Residual *Z*-score: standardized residual terms of a linear regression of epigenetic against chronological age.

**Figure 3.**
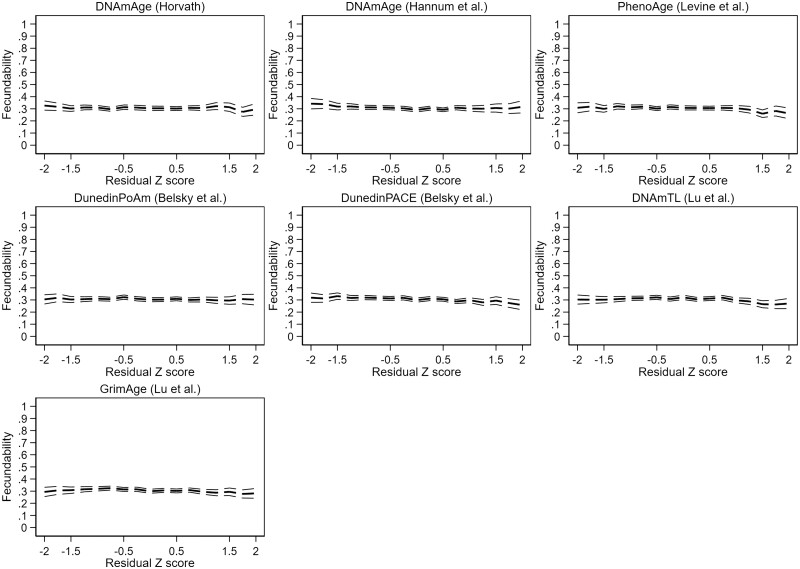
**Adjusted couple fecundability according to female epigenetic aging profile.** Fecundability ratio (FR) adjusted for pre-pregnancy body mass index, smoking, and highest completed or ongoing education. Residual *Z*-score: standardized residual terms of a linear regression of epigenetic against chronological age.

We found evidence for significant non-linearity in two of the models, DNAmAge by Hannum and PhenoAge. In the categorical analyses of these models (Supplementary Table S4), we observed increased fecundability in the very accelerated aging category (*Z*-score >1.5) in the DNAmAge by Hannum model (FR 1.25 [1.03–1.51]) and increased fecundability both in the moderately decelerated (*Z*-score −1.5 to −0.5) and the moderately accelerated (*Z*-score 0.5–1.5) aging categories in the PhenoAge model (respective FRs 1.13 [1.00–1.26] and 1.13 [1.01–1.21]). Results are represented graphically in Fig. 2 (unadjusted results) and Fig. 3 (adjusted results).

### Men

The unadjusted linear estimate from the DNAmAge by Hannum model indicated a slight linear decrease in fecundability with increasing EAA, with an FRs of 0.95 (0.91–1.00). This estimate was not robust to adjustment for covariates (Table 3). Additionally, the adjusted result from the DunedinPACE model demonstrated a slight linear increase in fecundability with increasing epigenetic age (FR 1.05 [1.00–1.10]. This corresponds to a 5% increase in the monthly probability of conception per standard deviation increase in EAA. This result was not robust when evaluated at an FDR of 5% (Supplementary Table S5).

**Table 3.** Unadjusted and adjusted fecundability ratios according to male epigenetic aging.

		Fecundability ratio	95% confidence interval	*P*, linear trend	*P*, test of non-linearity
**DNAmAge (Horvath)**	**Unadjusted**	0.97	0.92–1.01	0.144	**<0.001**
	**Adjusted**	0.97	0.92–1.01	0.131	**<0.001**
**DNAmAge (Hannum *et al.*)**	**Unadjusted**	**0.95**	**0.91–1.00**	**0.046**	**<0.001**
	**Adjusted**	0.96	0.92–1.01	0.091	**<0.001**
**PhenoAge (Levine *et al.*)**	**Unadjusted**	0.96	0.92–1.00	0.075	**<0.001**
	**Adjusted**	0.96	0.92–1.01	0.120	**<0.001**
**DunedinPoAm (Belsky *et al.*)**	**Unadjusted**	0.98	0.94–1.02	0.331	0.290
	**Adjusted**	0.99	0.94–1.04	0.648	0.332
**DunedinPACE (Belsky *et al.*)**	**Unadjusted**	1.02	0.98–1.07	0.311	0.460
	**Adjusted**	**1.05**	**1.00–1.10**	**0.037**	0.535
**DNAmTL (Lu *et al.*)**	**Unadjusted**	0.99	0.95–1.04	0.728	0.600
	**Adjusted**	0.99	0.94–1.03	0.557	0.622
**GrimAge (Lu *et al.*)**	**Unadjusted**	0.97	0.93–1.02	0.248	0.412
	**Adjusted**	0.98	0.93–1.04	0.560	0.362

Adjusted for body mass index, smoking, and highest completed or ongoing education. Fecundability ratios per one standard deviation increase in epigenetic age acceleration.Statistically significant results at *α* = 0.05 are highlighted in bold.

**Figure 4.**
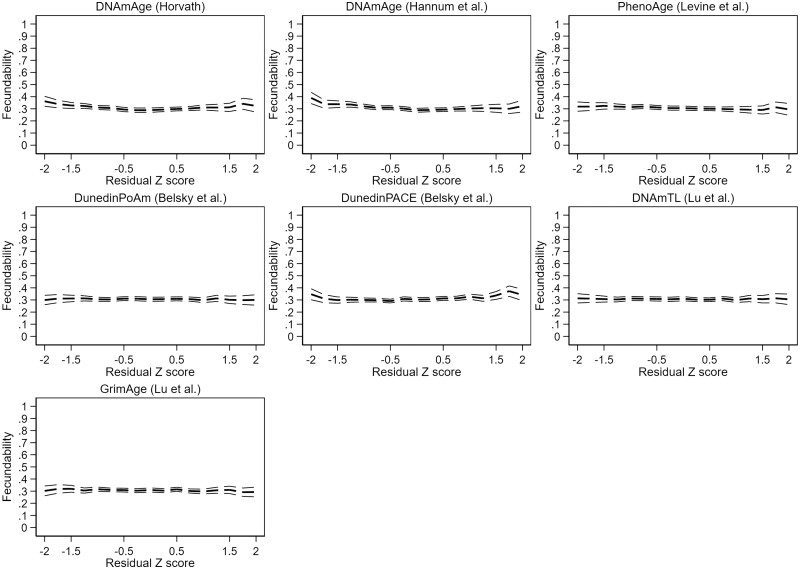
**Unadjusted couple fecundability according to male epigenetic aging profile.** Residual *Z*-score: standardized residual terms of a linear regression of epigenetic against chronological age.

**Figure 5.**
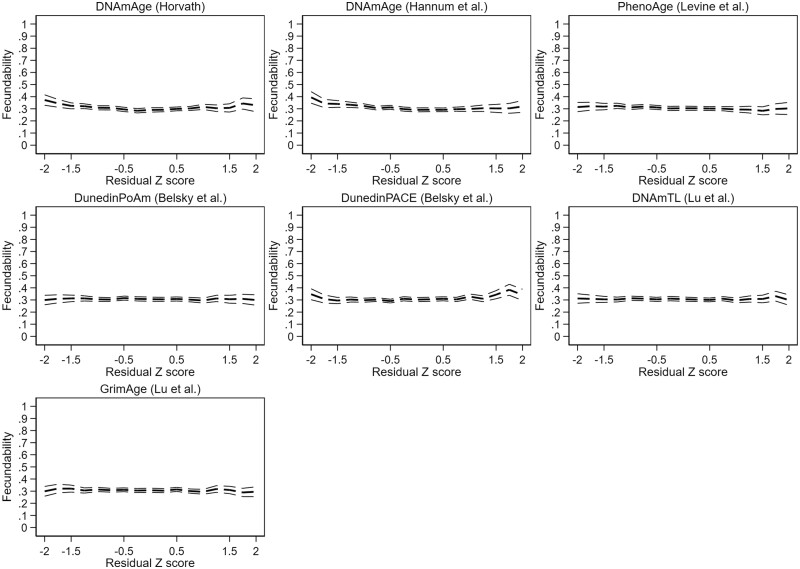
**Adjusted couple fecundability according to male epigenetic aging profile.** Fecundability ratio (FR) adjusted for body mass index, smoking, and highest completed or ongoing education. Residual *Z*-score: standardized residual terms of a linear regression of epigenetic against chronological age.

There was evidence for significant non-linearity in the adjusted DNAmAge by Horvath, DNAmAge by Hannum and PhenoAge models. In the categorical models, the only robust finding was increased fecundability at very decelerated epigenetic aging (*Z*-score <−1.5) in the DNAmAge by Horvath model (FR 1.23 [1.03–1.47]), as shown in Supplementary Table S6. Results are represented graphically in Fig. 4 (unadjusted results) and Fig. 5 (adjusted results).

### Sensitivity analyses

The couple-level sensitivity analysis did not demonstrate any notable patterns suggesting a pronounced effect of different partner epigenetic profile combinations (Supplementary Table S7). In women, the epigenetic age differences in fecundability did not vary between women <30 and women ≥30 (Supplementary Table S8). In men, the trend towards higher fecundability with increased epigenetic aging that was observed in the DunedinPACE model in the main analysis was only significant in the <30 group, suggesting that chronologically younger men are driving this trend in the main analysis (Supplementary Table S9). Additionally, there was a trend toward lower fecundability with increased epigenetic aging in men ≥30 in the Horvath model (FR 0.94, 95% CI 0.83–1.00) that was not seen among men <30. However, this trend had only borderline significance (*P* = 0.045). Additional adjustment for parity, chronological age, and blood sample cell type composition, as well as the inclusion of non-planners, yielded similar results to the main analysis (Supplementary Tables S10 and S11).

## Discussion

In this population-based pregnancy cohort, we investigated the association between DNA-methylation based EAA/EAD and fecundability among men and women with naturally conceived pregnancies. Overall, we observed no clear differences in fecundability according to EAA/EAD in women or men when examining linear relationships and accounting for the effects of multiple testing. Considering isolated models, there were only indications of weak associations with magnitudes of 4–5% differences in the probability of conception per standard deviation increase in EAA. Some indications of non-linear associations were also observed, but estimates for differences in fecundability according to categories of epigenetic age were both weak and heterogeneous between the seven epigenetic clocks. Non-linear relationships should be further explored in other datasets.

We have adjusted for factors known to influence fecundity, and provide both crude and adjusted estimates. Both smoking and BMI have also been shown to influence EAA (Cardenas *et al.*, 2022; Klopack *et al.*, 2022; Lundgren *et al.*, 2022). Such variables are commonly adjusted for in research into epigenetic aging. However, they can also be argued to be an integral part of biological aging, complicating the evaluation of estimates from adjusted models. Interestingly, we did not see very different results with adjustment, further supporting a lack of association between EAA and fecundability in natural conception.

Our findings contrast those of several prior studies in females finding an association between accelerated epigenetic aging and unfavorable clinical fecundity outcomes (Hanson *et al.*, 2020; Monseur *et al.*, 2020; Knight *et al.*, 2022; Li Piani *et al.*, 2022) and use of IVF (Lee *et al.*, 2022). They also contrast with prior studies in males reporting associations between sperm-based EAD and shorter TTPs, and between higher global sperm DNA methylation and higher sperm count, concentration and motility and higher pregnancy rates after ICSI (Roshdy *et al.*, 2020; Pilsner *et al.*, 2022). A possible explanation for these contrasts is that our study focuses on couples who conceived naturally. The discrepancy between our findings and those of prior studies may therefore reflect that epigenetic aging effects are absent or very subtle among couples who conceive naturally, and more pronounced in those seeking medical assistance for infertility. A recent meta-analysis (Peigné *et al.*, 2023) showed that AMH, which is a marker for ovarian reserve, was linked to live birth rates after ART, but found few studies in non-ART populations. However, summarizing the available data on AMH-based prediction of clinical pregnancy, TTP and fecundability, the authors conclude that the ‘evidence seems reasonably strong that AMH cannot be used to predict fertility potential in the general population’. Whilst more research is clearly needed, the available evidence could suggest that diminished ovarian reserves marked by low AMH do not markedly impair natural conception rates. As such, any effects of EAA on ovarian reserve might not have clinically meaningful implications in natural conception cohorts.

Another possible explanation for the contrast between our results and prior studies is that fecundability is a couple-level measure, so that the direct impacts of epigenetic aging in each partner may be obscured by effects of the other partner. We attempted to explore this in a sensitivity analysis grouping couples according to the combined epigenetic aging profile of males and females, but were unable to uncover any relevant effects, possibly due to the groups being too small. An interesting avenue for further research would be exploring the association between epigenetic aging and partner-specific fecundity parameters, such as semen quality or AMH, in non-ART populations.

Important strengths of our study include the large sample size and the availability of detailed information on TTP from a non-ART population. TTP has been shown to be well recalled when reported retrospectively during pregnancy (Radin *et al.*, 2015). As all participants in our study conceived naturally, we are also able to exclude the possibility of reverse causation due to age-accelerating effects of ART, which is an issue when investigating this association in an ART population.

A limitation of our study is the temporal gap between the time when the couple started trying to conceive and the time of achievement of pregnancy. One consequence of this is that couples experiencing prolonged conception times are older at the time of blood sample collection and questionnaire administration. We addressed this limitation by censoring data at 6 months. As the pace of epigenetic aging is assumed to be relatively constant over time (Mareckova *et al.*, 2023), it is reasonable to use epigenetic aging in week 18 of the pregnancy as a proxy for epigenetic aging at the initiation of conception attempts.

There is also risk of selection bias, both given the low overall participation rate in MoBa (41%) and because participants in MoBa with available blood samples have higher education, lower smoking rates and higher rates of infertility (Hernáez *et al.*, 2024). Additionally, MoBa is a pregnancy cohort, including only participants with pregnancies lasting beyond the first trimester. This may reduce the generalizability of our results, e.g. to couples who are sterile or experience early miscarriages.

As delayed childbearing becomes more common, understanding the mechanisms behind the age-related decline in fecundity in both women and men is of increasing importance. In this study, we did not find compelling evidence of an association between epigenetic aging and fecundability in couples with naturally conceived pregnancies. The lack of an association between EAA/EAD and fecundability in couples with naturally conceived pregnancies is an important and clinically relevant finding, as it suggests that factors other than accelerated epigenetic aging drive the age-related decline in fecundity among couples who conceive naturally. If future studies corroborate this, it may also enhance our understanding of how couples with and without infertility differ, given the potential role of epigenetic aging demonstrated in ART populations (Hanson *et al.*, 2020; Monseur *et al.*, 2020; Roshdy *et al.*, 2020; Knight *et al.*, 2022; Lee *et al.*, 2022; Li Piani *et al.*, 2022; Pilsner *et al.*, 2022). This insight could lead to more targeted approaches in fertility research and treatment.

## Supplementary Material

deae242_Supplementary_Figure_S1

deae242_Supplementary_Figure_S2

deae242_Supplementary_Table_S1

deae242_Supplementary_Table_S2

deae242_Supplementary_Table_S3

deae242_Supplementary_Table_S4

deae242_Supplementary_Table_S5

deae242_Supplementary_Table_S6

deae242_Supplementary_Table_S7

deae242_Supplementary_Table_S8

deae242_Supplementary_Table_S9

deae242_Supplementary_Table_S10

deae242_Supplementary_Table_S11

## Data Availability

The data used for this paper are available by application to the Norwegian Mother, Father and Child Cohort study (mobaadm@fhi.no) and by application to www.helsedata.no. Ethical approval is required.
