# Supplementary material for: Epigenetic aging and fecundability: the Norwegian Mother, Father and Child Cohort Study
Source: Hum Reprod. 2024 Oct 22;39(12):2806–15. doi: 10.1093/humrep/deae242 (PMC11630011; doi:10.1093/humrep/deae242)
Supplement: deae242_Supplementary_Table_S1 [file deae242_supplementary_table_s1.pdf]

OXFORD

human reproduction

*Human Reproduction*, 2024, 1–1

https://doi.org/10.1093/humrep/deae242

Supplementary Data

**Supplementary Table S1.** Characteristics of all men and women from complete mother-father-child trios.

| Characteristics | Women | Men |
|---|---|---|
| **Age, mean (SD)** | 30.2 (4.5) | 32.7 (5.3) |
| **Education (%)** | | |
| Less than high school | 5.4 | 7.4 |
| High school | 26.0 | 36.7 |
| College, up to 4 years | 41.7 | 27.8 |
| College, more than 4 years | 26.4 | 25.0 |
| Missing | 0.5 | 3.0 |
| **Smoking (%)** | | |
| No | 72.3 | 69.7 |
| Sometimes | 10.0 | 11.5 |
| Daily | 16.9 | 18.8 |
| Missing | 0.9 | 0.0 |
| **Body mass index (pre-pregnancy for women) (%)** | | |
| Underweight (<18.5) | 3.0 | 0.2 |
| Normal weight (18.5–24.9) | 63.7 | 42.5 |
| Overweight (25–29.9) | 21.3 | 43.5 |
| Obese (≥30) | 9.4 | 9.8 |
| Missing | 2.7 | 4.0 |
| **Parity (%)** | | |
| 0 | 49.1 | |
| 1 | 34.7 | |
| 2 | 13.4 | |
| ≥3 | 2.8 | |
