# Supplementary material for: Epigenetic aging and fecundability: the Norwegian Mother, Father and Child Cohort Study
Source: Hum Reprod. 2024 Oct 22;39(12):2806–15. doi: 10.1093/humrep/deae242 (PMC11630011; doi:10.1093/humrep/deae242)
Supplement: deae242_Supplementary_Table_S2 [file deae242_supplementary_table_s2.pdf]

Human Reproduction, 2024, 1–1
https://doi.org/10.1093/humrep/deae242
Supplementary Data

Supplementary Table S2. Pairwise Pearson correlation coefficients of the seven epigenetic clocks, women.

	DNAmAge (Horvath)	DNAmAge (Hannum *et al.*)	PhenoAge (Levine *et al.*)	DunedinPoAm (Belsky *et al.*)	DunedinPACE (Belsky *et al.*)	DNAmTL (Lu *et al.*)	GrimAge (Lu *et al.*)
**DNAmAge (Horvath)**	1.000						
**DNAmAge (Hannum *et al.*)**	0.452	1.000					
**PhenoAge (Levine *et al.*)**	0.693	0.708	1.000				
**DunedinPoAm (Belsky *et al.*)**	0.454	0.067	0.407	1.000			
**DunedinPACE (Belsky *et al.*)**	0.057	0.197	0.337	0.502	1.000		
**DNAmTL (Lu *et al.*)**	−0.178	−0.593	−0.469	−0.035	−0.254	1.000	
**GrimAge (Lu *et al.*)**	0.588	0.653	0.735	0.478	0.406	−0.517	1.000
