# Supplementary material for: Epigenetic aging and fecundability: the Norwegian Mother, Father and Child Cohort Study
Source: Hum Reprod. 2024 Oct 22;39(12):2806–15. doi: 10.1093/humrep/deae242 (PMC11630011; doi:10.1093/humrep/deae242)
Supplement: deae242_Supplementary_Table_S3 [file deae242_supplementary_table_s3.pdf]

OXFORD

human reproduction

Human Reproduction, 2024, 1–1
https://doi.org/10.1093/humrep/deae242
Supplementary Data

Supplementary Table S3. Pairwise Pearson correlation coefficients of the seven epigenetic clocks, men.

	DNAmAge (Horvath)	DNAmAge (Hannum *et al.*)	PhenoAge (Levine *et al.*)	DunedinPoAm (Belsky *et al.*)	DunedinPACE (Belsky *et al.*)	DNAmTL (Lu *et al.*)	GrimAge (Lu *et al.*)
**DNAmAge (Horvath)**	1.000						
**DNAmAge (Hannum *et al.*)**	0.497	1.000					
**PhenoAge (Levine *et al.*)**	0.738	0.745	1.000				
**DunedinPoAm (Belsky *et al.*)**	0.440	0.085	0.411	1.000			
**DunedinPACE (Belsky *et al.*)**	0.102	0.250	0.346	0.513	1.000		
**DNAmTL (Lu *et al.*)**	−0.258	−0.657	−0.553	−0.104	−0.344	1.000	
**GrimAge (Lu *et al.*)**	0.599	0.763	0.763	0.531	0.610	−0.567	1.000
