# Supplementary material for: Epigenetic aging and fecundability: the Norwegian Mother, Father and Child Cohort Study
Source: Hum Reprod. 2024 Oct 22;39(12):2806–15. doi: 10.1093/humrep/deae242 (PMC11630011; doi:10.1093/humrep/deae242)
Supplement: deae242_Supplementary_Table_S4 [file deae242_supplementary_table_s4.pdf]

OXFORD
human reproduction

Human Reproduction, 2024, 1–1
https://doi.org/10.1093/humrep/deae242
Supplementary Data

Supplementary Table S4. Fecundability ratios, 95% confidence intervals and *P*-values according to female epigenetic age acceleration.

		Epigenetic age acceleration category				
		**Very decelerated aging (Z <−1.5)**	**Moderately decelerated aging (Z = −1.5–0.5)**	**Reference, neither decelerated nor accelerated aging (Z = −0.5–0.5)**	**Moderately accelerated aging (Z = 0.5–1.5)**	**Very accelerated aging (Z > 1.5)**
**DNAmAge (Horvath)**	**Unadjusted**	1.05 (0.88–1.26)	0.95 (0.84–1.08)	1	0.98 (0.88–1.10)	0.85 (0.67–1.08)
	P	0.564	0.443		0.722	0.189
	**Adjusted**[1]	1.05 (0.88–1.26)	0.96 (0.85–1.09)	1	0.98 (0.87–1.09)	0.88 (0.70–1.13)
	P	0.563	0.543		0.662	0.317
**DNAmAge (Hannum *et al.*)**	**Unadjusted**	1.10 (0.91–1.34)	1.05 (0.94–1.18)	1	0.96 (0.85–1.07)	1.19 (0.98–1.44)
	P	0.320	0.401		0.440	0.085
	**Adjusted**[1]	1.05 (0.86–1.27)	1.05 (0.94–1.18)	1	0.95 (0.85–1.07)	**1.22 (1.00–1.48)**
	P	0.650	0.366		0.430	**0.046**
**PhenoAge (Levine *et al.*)**	**Unadjusted**	1.14 (0.94–1.38)	**1.13 (1.01–1.27)**	1	**1.13 (1.01–1.27)**	0.80 (0.62–1.02)
	P	0.196	**0.038**		**0.028**	0.075
	**Adjusted**[1]	1.09 (0.89–1.33)	**1.13 (1.00–1.26)**	1	**1.13 (1.01–1.27)**	0.84 (0.65–1.08)
	P	0.307	**0.049**		**0.027**	0.171
**DunedinPoAm (Belsky *et al.*)**	**Unadjusted**	1.05 (0.87–1.26)	0.97 (0.87–1.09)	1	0.98 (0.87–1.10)	0.82 (0.66–1.02)
	P	0.609	0.646		0.756	0.074
	**Adjusted**[1]	1.03 (0.86–1.24)	0.97 (0.86–1.08)	1	1.00 (0.89–1.13)	0.84 (0.68–1.05)
	P	0.740	0.555		0.967	0.134
**DunedinPACE (Belsky *et al.*)**	**Unadjusted**	0.97 (0.80–1.17)	1.07 (0.95–1.19)	1	0.93 (0.82–1.04)	0.84 (0.69–1.02)
	P	0.734	0.272		0.211	0.084
	**Adjusted**[1]	0.92 (0.76–0.12)	1.04 (0.93–1.17)	1	0.96 (0.85–1.08)	0.93 (0.76–1.13)
	P	0.429	0.452		0.520	0.464
**DNAmTL (Lu *et al.*)**	**Unadjusted**	0.97 (0.80–1.18)	1.02 (0.91–1.15)	1	1.07 (0.96–1.20)	**0.80 (0.65–0.99)**
	P	0.779	0.696		0.225	**0.039**
	**Adjusted**[1]	0.97 (0.80–1.18)	1.04 (0.92–1.17)	1	1.05 (0.93–1.17)	**0.79 (0.64–0.98)**
	P	0.765	0.527		0.436	**0.029**
**GrimAge (Lu *et al.*)**	**Unadjusted**	1.01 (0.89–1.24)	**1.12 (1.00–1.25)**	1	1.10 (0.98–1.24)	**0.79 (0.64–0.98)**
	P	0.900	**0.047**		0.111	**0.028**
	**Adjusted**[1]	0.98 (0.80–1.20)	1.11 (0.99–1.24)	1	1.12 (0.99–1.26)	0.81 (0.64–1.02)
	P	0.878	0.063		0.079	0.068

[1] Adjusted for pre-pregnancy body mass index, smoking, and highest completed or ongoing education.
Statistically significant results at $\alpha = 0.05$ are highlighted in bold.
