# Supplementary material for: Epigenetic aging and fecundability: the Norwegian Mother, Father and Child Cohort Study
Source: Hum Reprod. 2024 Oct 22;39(12):2806–15. doi: 10.1093/humrep/deae242 (PMC11630011; doi:10.1093/humrep/deae242)
Supplement: deae242_Supplementary_Table_S5 [file deae242_supplementary_table_s5.pdf]

Human Reproduction, 2024, 1–1
https://doi.org/10.1093/humrep/deae242
Supplementary Data

Supplementary Table S5. Likelihood ratio test of linear models for men.

Men	P-value of adjusted linear model	Rank	Benjamini Hochberg corrected α	Significant (yes/no)
**DNAmAge (Horvath)**	0.131	4	0.029	No
**DNAmAge (Hannum *et al.*)**	0.091	2	0.014	No
**PhenoAge (Levine *et al.*)**	0.120	3	0.021	No
**DunedinPoAm (Belsky *et al.*)**	0.648	7	0.050	No
**DunedinPACE (Belsky *et al.*)**	0.037	1	0.007	No
**DNAmTL (Lu *et al.*)**	0.557	5	0.036	No
**GrimAge (Lu *et al.*)**	0.560	6	0.043	No

Models for women omitted, as there were no significant results at $\alpha = 0.05$.
