# Supplementary material for: Epigenetic aging and fecundability: the Norwegian Mother, Father and Child Cohort Study
Source: Hum Reprod. 2024 Oct 22;39(12):2806–15. doi: 10.1093/humrep/deae242 (PMC11630011; doi:10.1093/humrep/deae242)
Supplement: deae242_Supplementary_Table_S6 [file deae242_supplementary_table_s6.pdf]

OXFORD
human reproduction

Human Reproduction, 2024, 1–1
https://doi.org/10.1093/humrep/deae242
Supplementary Data

Supplementary Table S6. Fecundability ratios, 95% confidence intervals, and *P*-values according to male epigenetic age acceleration.

		Epigenetic age acceleration category				
		**Very decelerated aging (Z <−1.5)**	**Moderately decelerated aging (Z = −1.5–0.5)**	**Reference, neither decelerated nor accelerated aging (Z = −0.5–0.5)**	**Moderately accelerated aging (Z = 0.5–1.5)**	**Very accelerated aging (Z > 1.5)**
**DNAmAge (Horvath)**	**Unadjusted**	**1.23 (1.03–1.47)**	1.10 (0.98–1.25)	1	1.06 (0.95–1.19)	1.04 (0.81–1.34)
	P	**0.023**	0.104		0.321	0.739
	**Adjusted**[1]	**1.23 (1.03–1.47)**	1.12 (1.00–1.27)	1	1.07 (0.95–1.20)	1.06 (0.83–1.36)
	P	**0.021**	0.057		0.248	0.651
**DNAmAge (Hannum *et al.*)**	**Unadjusted**	1.20 (0.99–1.44)	1.09 (0.97–1.23)	1	1.01 (0.90–1.14)	1.12 (0.92–1.36)
	P	0.073	0.173		0.693	0.353
	**Adjusted**[1]	1.17 (0.97–1.41)	1.08 (0.96–1.21)	1	1.01 (0.90–1.14)	1.11 (0.91–1.35)
	P	.101	.223		.818	.297
**PhenoAge (Levine *et al.*)**	**Unadjusted**	1.05 (0.87–1.26)	1.05 (0.93–1.18)	1	0.93 (0.83–1.04)	0.99 (0.79–1.22)
	P	0.614	0.401		0.185	0.897
	**Adjusted**[1]	1.02 (0.85–1.22)	1.05 (0.93–1.18)	1	0.92 (0.82–1.03)	1.00 (0.81–1.24)
	P	0.834	0.437		0.159	0.997
**DunedinPoAm (Belsky *et al.*)**	**Unadjusted**	0.92 (0.74–1.14)	1.01 (0.90–1.13)	1	0.9 (0.88–1.12)	0.89 (0.74–1.08)
	P	0.454	0.893		0.863	0.251
	**Adjusted**[1]	0.93 (0.75–1.16)	1.00 (0.90–1.12)	1	1.02 (0.90–1.15)	0.92 (0.75–1.13)
	P	0.520	0.943		0.765	0.421
**DunedinPACE (Belsky *et al.*)**	**Unadjusted**	1.14 (0.94–1.40)	0.97 (0.86–1.08)	1	1.02 (0.90–1.15)	1.11 (0.93–1.33)
	P	0.187	0.564		0.787	0.233
	**Adjusted**[1]	1.11 (0.91–1.35)	0.93 (0.83–1.04)	1	1.05 (0.93–1.19)	1.18 (0.98–1.41)
	P	0.325	0.222		0.408	0.086
**DNAmTL (Lu *et al.*)**	**Unadjusted**	0.94 (0.78–1.13)	1.02 (0.91–1.15)	1	0.95 (0.85–1.07)	1.00 (0.82–1.21)
	P	0.509	0.707		0.400	0.976
	**Adjusted**[1]	0.94 (0.78–1.14)	1.02 (0.91–1.15)	1	0.95 (0.85–1.07)	0.96 (0.79–1.17)
	P	0.524	0.681		0.385	0.671
**GrimAge (Lu *et al.*)**	**Unadjusted**	0.95 (0.77–1.19)	1.02 (0.91–1.13)	1	0.97 (0.85–1.10)	0.87 (0.72–1.04)
	P	0.683	0.783		0.646	0.122
	**Adjusted**[1]	0.94 (0.76–1.17)	1.01 (0.91–1.13)	1	0.86 (0.85–1.12)	0.87 (0.71–1.08)
	P	0.595	0.852		0.738	0.185

[1] Adjusted for body mass index, smoking, and highest completed or ongoing education.
Statistically significant results at $\alpha = 0.05$ are highlighted in bold.
