# Supplementary material for: Epigenetic aging and fecundability: the Norwegian Mother, Father and Child Cohort Study
Source: Hum Reprod. 2024 Oct 22;39(12):2806–15. doi: 10.1093/humrep/deae242 (PMC11630011; doi:10.1093/humrep/deae242)
Supplement: deae242_Supplementary_Table_S7 [file deae242_supplementary_table_s7.pdf]

OXFORD

human reproduction

Human Reproduction, 2024, 1–1

https://doi.org/10.1093/humrep/deae242

Supplementary Data

Supplementary Table S7. Adjusted fecundability ratios according to combinations of epigenetic aging profiles for both partners in each couple.

		Neither partner EAA/EAD[1]	Woman EAA, man neither	Woman neither, man EAA	Both partners EAA	Woman EAD, man neither	Woman neither, man EAD	Both partners EAD	Woman EAA, man EAD	Woman EAD, man EAA
**DNAmAge (Horvath)**	**FR**	1	0.94	0.99	0.95	**0.78**	0.98	1.06	1.12	0.86
	**CI**	–	0.78–1.14	0.82–1.19	0.81–1.15	**0.64–0.97**	0.80–1.21	0.91–1.25	0.74–1.71	0.51–1.46
	**N**	183	185	198	399	149	127	381	20	15
**DNAmAge (Hannum *et al.*)**	**FR**	1	1.04	1.08	1.10	1.18	**1.26**	1.10	1.10	1.14
	**CI**	–	0.88–1.25	0.90–1.28	0.92–1.31	1.00–1.40	**1.06–1.49**	0.92–1.32	0.89–1.34	0.93–1.40
	**N**	284	200	193	187	201	185	174	116	117
**PhenoAge (Levine *et al.*)**	**FR**	1	1.00	0.87	1.02	1.04	0.98	1.11	1.13	1.00
	**CI**	–	0.85–1.18	0.73–1.03	0.86–1.21	0.88–1.24	0.82–1.16	0.94–1.32	0.92–1.39	0.82–1.22
	**N**	285	225	212	181	178	189	171	97	119
**DunedinPoAm (Belsky *et al.*)**	**FR**	1	1.01	0.97	0.97	0.96	1.02	0.98	0.90	1.06
	**CI**	–	0.85–1.19	0.81–1.18	0.82–1.15	0.80–1.14	0.86–1.21	0.84–1.14	0.69.1,.17	0.82–1.36
	**N**	277	204	163	250	168	172	296	65	62
**DunedinPACE (Belsky *et al.*)**	**FR**	1	1.03	1.16	1.03	1.01	0.96	1.01	0.88	1.17
	**CI**	–	0.86–1.22	0.97–1.38	0.85–1.24	0.86–1.20	0.81–1.14	0.85–1.20	0.71–1.09	0.96–1.43
	**N**	262	206	174	180	209	23	189	116	108
**DNAmTL (Lu *et al.*)**	**FR**	1	1.08	0.98	0.93	1.06	1.09	0.99	1.04	1.11
	**CI**	–	0.91–1.29	0.82–1.17	0.77–1.11	.88–1.28	0.91–1.30	0.82–1.19	0.85–1.28	0.91–1.34
	**N**	219	212	208	190	186	195	181	125	141
**GrimAge (Lu *et al.*)**	**FR**	1	1.06	0.84	1.00	1.00	1.01	1.04	0.93	1.14
	**CI**	–	0.89–1.25	0.69–1.02	0.82–1.22	0.84–1.18	0.85–1.19	0.89–1.21	0.73–1.17	0.92–1.42
	**N**	292	215	169	173	192	193	254	84	85

Adjusted for female pre-pregnancy body mass index, male body mass index, male and female smoking, and male and female highest completed or ongoing education.

[1] EAA, epigenetic age acceleration; EAD, epigenetic age deceleration. For this particular model, EAA and EAD were defined as having Z-scores (standardized residual term of a regression of epigenetic age against chronological age) >0.5 and <–0.5 respectively, as the thresholds of >1.5 and <–1.5 used in the main analyses yielded too small groups.

Statistically significant results at $\alpha = 0.05$ are highlighted in bold.

FR, fecundability ratio; CI, confidence interval.
