# Supplementary material for: Epigenetic aging and fecundability: the Norwegian Mother, Father and Child Cohort Study
Source: Hum Reprod. 2024 Oct 22;39(12):2806–15. doi: 10.1093/humrep/deae242 (PMC11630011; doi:10.1093/humrep/deae242)
Supplement: deae242_Supplementary_Table_S8 [file deae242_supplementary_table_s8.pdf]

OXFORD
human reproduction

Human Reproduction, 2024, 1–1
https://doi.org/10.1093/humrep/deae242
Supplementary Data

Supplementary Table S8. Adjusted couple fecundability according to female epigenetic aging profile, stratified by female chronological age.

	Chronological age	Fecundability ratio	95% confidence interval	P
**DNAmAge (Horvath)**	<30	0.98	0.92–1.04	0.542
	≥30	0.99	0.93–1.06	0.739
**DNAmAge (Hannum *et al.*)**	<30	0.98	0.91–1.04	0.484
	≥30	1.00	0.93–1.07	0.963
**PhenoAge (Levine *et al.*)**	<30	0.95	0.88–1.01	0.099
	≥30	0.99	0.92–1.05	0.660
**DunedinPoAm (Belsky *et al.*)**	<30	0.97	0.91–1.04	0.419
	≥30	0.99	0.92–1.06	0.696
**DunedinPACE (Belsky *et al.*)**	<30	0.95	0.89–1.02	0.149
	≥30	1.00	0.94–1.07	0.914
**DNAmTL (Lu *et al.*)**	<30	1.01	0.94–1.08	0.779
	≥30	0.95	0.89–1.00	0.068
**GrimAge (Lu *et al.*)**	<30	0.94	0.87–1.00	0.065
	≥30	1.00	0.93–1.00	0.981

Adjusted for body mass index, smoking, and highest completed or ongoing education. Fecundability ratios per one standard deviation increase in epigenetic age acceleration.
