# Supplementary material for: Epigenetic aging and fecundability: the Norwegian Mother, Father and Child Cohort Study
Source: Hum Reprod. 2024 Oct 22;39(12):2806–15. doi: 10.1093/humrep/deae242 (PMC11630011; doi:10.1093/humrep/deae242)
Supplement: deae242_Supplementary_Table_S9 [file deae242_supplementary_table_s9.pdf]

OXFORD

human reproduction

Human Reproduction, 2024, 1–1
https://doi.org/10.1093/humrep/deae242
Supplementary Data

Supplementary Table S9. Adjusted couple fecundability according to male epigenetic aging profile, stratified by male chronological age.

	Chronological age	Fecundability ratio	95% confidence interval	P
**DNAmAge (Horvath)**	<30	1.01	0.93–1.10	0.755
	≥30	**0.94**	**0.83–1.00**	**0.045**
**DNAmAge (Hannum *et al.*)**	<30	0.99	0.91–1.08	0.834
	≥30	0.95	0.91–1.00	0.067
**PhenoAge (Levine *et al.*)**	<30	1.04	0.96–1.12	0.381
	≥30	**0.93**	**0.88–0.98**	**0.011**
**DunedinPoAm (Belsky *et al.*)**	<30	0.99	0.91–1.09	0.864
	≥30	0.98	0.93–1.04	0.578
**DunedinPACE (Belsky *et al.*)**	<30	**1.16**	**1.07–1.26**	**0.000**
	≥30	1.01	0.95–1.08	0.683
**DNAmTL (Lu *et al.*)**	<30	0.95	0.88–1.03	0.206
	≥30	1.00	0.95–1.06	0.921
**GrimAge (Lu *et al.*)**	<30	1.05	0.96–1.16	0.228
	≥30	0.96	0.90–1.02	0.161

Adjusted for body mass index, smoking, and highest completed or ongoing education. Fecundability ratios per one standard deviation increase in epigenetic age acceleration. Statistically significant results at $\alpha = 0.05$ are highlighted in bold.
