# Supplementary material for: Epigenetic aging and fecundability: the Norwegian Mother, Father and Child Cohort Study
Source: Hum Reprod. 2024 Oct 22;39(12):2806–15. doi: 10.1093/humrep/deae242 (PMC11630011; doi:10.1093/humrep/deae242)
Supplement: deae242_Supplementary_Table_S10 [file deae242_supplementary_table_s10.pdf]

OXFORD
human reproduction
Human Reproduction, 2024, 1–1
https://doi.org/10.1093/humrep/deae242
Supplementary Data

Supplementary Table S10. Adjusted couple fecundability according to female epigenetic aging profile with (i) additional adjustment for parity, (ii) additional adjustment for chronological age, (iii) additional adjustment for blood sample cell-type composition, and (iv) including non-planners in the study sample.

	Sensitivity analysis	Fecundability ratio	95% confidence interval	P
**DNAmAge (Horvath)**	Parity adjustment	0.99	0.94–1.03	0.528
	Chronological age adjustment	0.99	0.94–1.03	0.559
	Cell-type adjustment	0.96	0.87–1.06	0.394
	Non-planner inclusion	0.99	0.95–1.03	0.543
**DNAmAge (Hannum *et al.*)**	Parity adjustment	0.98	0.93–1.03	0.355
	Chronological age adjustment	0.99	0.94–1.04	0.612
	Cell-type adjustment	0.98	0.91–1.06	0.606
	Non-planner inclusion	0.98	0.94–1.02	0.300
**PhenoAge (Levine *et al.*)**	Parity adjustment	0.96	0.92–1.01	0.123
	Chronological age adjustment	0.97	0.92–1.01	0.178
	Cell-type adjustment	0.94	0.88–1.01	0.103
	Non-planner inclusion	0.99	0.95–1.03	0.512
**DunedinPoAm (Belsky *et al.*)**	Parity adjustment	0.98	0.93–1.03	0.386
	Chronological age adjustment	0.98	0.94–1.03	0.469
	Cell-type adjustment	0.97	0.88–1.07	0.591
	Non-planner inclusion	0.99	0.95–1.03	0.512
**DunedinPACE (Belsky *et al.*)**	Parity adjustment	0.98	0.93–1.03	0.364
	Chronological age adjustment	0.98	0.93–1.02	0.344
	Cell-type adjustment	0.95	0.88–1.03	0.185
	Non-planner inclusion	0.98	0.94–1.02	0.418
**DNAmTL (Lu *et al.*)**	Parity adjustment	0.98	0.94–1.02	0.340
	Chronological age adjustment	0.97	0.93–1.02	0.232
	Cell-type adjustment	1.02	0.95–1.09	0.574
	Non-planner inclusion	0.99	0.95–1.03	0.546
**GrimAge (Lu *et al.*)**	Parity adjustment	0.97	0.92–1.02	0.191
	Chronological age adjustment	0.97	0.92–1.02	0.206
	Cell-type adjustment	0.94	0.86–1.02	0.146
	Non-planner inclusion	0.97	0.93–1.01	0.194

Adjusted for body mass index, smoking, and highest completed or ongoing education. Fecundability ratios per one standard deviation increase in epigenetic age acceleration.
