# Supplementary material for: Epigenetic aging and fecundability: the Norwegian Mother, Father and Child Cohort Study
Source: Hum Reprod. 2024 Oct 22;39(12):2806–15. doi: 10.1093/humrep/deae242 (PMC11630011; doi:10.1093/humrep/deae242)
Supplement: deae242_Supplementary_Table_S11 [file deae242_supplementary_table_s11.pdf]

Human Reproduction, 2024, 1–1
https://doi.org/10.1093/humrep/deae242
Supplementary Data

Supplementary Table S11. Adjusted couple fecundability according to male epigenetic aging profile with (i) additional adjustment for chronological age, (ii) additional adjustment for blood sample cell-type composition, and (iii) including non-planners in the study sample.

	Chronological age	Fecundability ratio	95% confidence interval	P
**DNAmAge (Horvath)**	Chronological age adjustment	0.97	0.92–1.01	0.149
	Cell-type adjustment	0.94	0.88–1.01	0.083
	Non-planner inclusion	0.98	0.94–1.02	0.260
**DNAmAge (Hannum *et al.*)**	Chronological age adjustment	0.96	0.92–1.01	0.087
	Cell-type adjustment	**0.95**	**0.90–1.00**	**0.039**
	Non-planner inclusion	**0.96**	**0.92–1.00**	**0.038**
**PhenoAge (Levine *et al.*)**	Chronological age adjustment	0.97	0.92–1.01	0.151
	Cell-type adjustment	**0.94**	**0.89–0.99**	**0.032**
	Non-planner inclusion	0.97	0.93–1.01	0.131
**DunedinPoAm (Belsky *et al.*)**	Chronological age adjustment	0.99	0.94–1.04	0.676
	Cell-type adjustment	0.99	0.93–1.06	0.764
	Non-planner inclusion	1.00	0.96–1.05	0.890
**DunedinPACE (Belsky *et al.*)**	Chronological age adjustment	**1.06**	**1.01–1.11**	**0.022**
	Cell-type adjustment	1.05	1.00–1.11	0.057
	Non-planner inclusion	**1.05**	**1.01–1.10**	**0.020**
**DNAmTL (Lu *et al.*)**	Chronological age adjustment	0.98	0.94–1.03	0.502
	Cell-type adjustment	1.01	0.96–1.06	0.828
	Non-planner inclusion	1.00	0.96–1.04	0.902
**GrimAge (Lu *et al.*)**	Chronological age adjustment	0.99	0.94–1.04	0.714
	Cell-type adjustment	0.97	0.90–1.04	0.377
	Non-planner inclusion	0.99	0.94–1.03	0.573

Adjusted for body mass index, smoking, and highest completed or ongoing education. Fecundability ratios per one standard deviation increase in epigenetic age acceleration. Statistically significant results at $\alpha = 0.05$ are highlighted in bold.

© The Author(s) 2024. Published by Oxford University Press on behalf of European Society of Human Reproduction and Embryology.
This is an Open Access article distributed under the terms of the Creative Commons Attribution License (https://creativecommons.org/licenses/by/4.0/), which permits unrestricted reuse, distribution, and reproduction in any medium, provided the original work is properly cited.
Human Reproduction, 2024, 00, 1–23
https://doi.org/10.1093/humrep/deae242
Original Article
